# Analyzing the gene expression profile of pediatric acute myeloid leukemia with real-time PCR arrays

**DOI:** 10.1186/1475-2867-12-40

**Published:** 2012-09-08

**Authors:** Tao Yan-Fang, Wu Dong, Pang Li, Zhao Wen-Li, Lu Jun, Wang Na, Wang Jian, Feng Xing, Li Yan-Hong, Ni Jian, Pan Jian

**Affiliations:** 1 Department of Hematology and Oncology, Children's Hospital of Soochow University, Suzhou, China; 2 Translational Research Center, Second Hospital, The Second Clinical School, Nanjing Medical University, Nanjing, China

**Keywords:** Pediatric, Acute myeloid leukemia, Real-time PCR array

## Abstract

**Background:**

The Real-time PCR Array System is the ideal tool for analyzing the expression of a focused panel of genes. In this study, we will analyze the gene expression profile of pediatric acute myeloid leukemia with real-time PCR arrays.

**Methods:**

Real-time PCR array was designed and tested firstly. Then gene expression profile of 11 pediatric AML and 10 normal controls was analyzed with real-time PCR arrays. We analyzed the expression data with MEV (Multi Experiment View) cluster software. Datasets representing genes with altered expression profile derived from cluster analyses were imported into the Ingenuity Pathway Analysis Tool.

**Results:**

We designed and tested 88 real-time PCR primer pairs for a quantitative gene expression analysis of key genes involved in pediatric AML. The gene expression profile of pediatric AML is significantly different from normal control; there are 19 genes up-regulated and 25 genes down-regulated in pediatric AML. To investigate possible biological interactions of differently regulated genes, datasets representing genes with altered expression profile were imported into the Ingenuity Pathway Analysis Tool. The results revealed 12 significant networks. Of these networks, Cellular Development, Cellular Growth and Proliferation, Tumor Morphology was the highest rated network with 36 focus molecules and the significance score of 41. The IPA analysis also groups the differentially expressed genes into biological mechanisms that are related to hematological disease, cell death, cell growth and hematological system development. In the top canonical pathways, p53 and Huntington’s disease signaling came out to be the top two most significant pathways with a p value of 1.5E-8 and2.95E-7, respectively.

**Conclusions:**

The present study demonstrates the gene expression profile of pediatric AML is significantly different from normal control; there are 19 genes up-regulated and 25 genes down-regulated in pediatric AML. We found some genes dyes-regulated in pediatric AML for the first time as FASLG, HDAC4, HDAC7 and some HOX family genes. IPA analysis showed the top important pathways for pediatric AML are p53 and Huntington’s disease signaling. This work may provide new clues of molecular mechanism in pediatric AML.

## Background

Pediatric acute myeloid leukemia (AML) comprises up to 20% of all childhood leukemia. Pediatric AML is a heterogeneous clonal disorder of hematopoietic progenitor cells, which lose the ability to differentiate normally and to respond to normal regulators of proliferation [1,2]. Gene microarray technology provides a powerful tool for characterizing gene expression on a genome scale [3-6]. Both cDNA and oligonucleotide-spotted microarrays have been used to find genes discriminative for the different genetic subgroups of pediatric AML [7]. Most reproducible and extensive results have been obtained using Affymetrix Gene Chips since these microarrays contain multiple perfect matches and mismatch oligonucleotides per gene and have been thoroughly validated. These studies in pediatric AML revealed new insights into the underlying biology of the different leukemic subtypes which may point to novel ways to treat these leukemia more specifically [8-10].

While microarray has been widely used in discovery-based medical and basic biological research, its direct application in clinical practice and regulatory decision-making has been questioned [11-13]. A few key issues, including the reproducibility, reliability, compatibility and standardization of microarray analysis and results, must be critically addressed before any routine usage of microarrays in clinical laboratory and regulated areas. However, in the absence of a "gold standard" or common reference for gene expression measurements, these evaluations and comparisons have often yield subjective and conflicting conclusions [14-18].

Real-time PCR is widely considered the gold standard for gene expression measurement due to its high assay specificity, high detection sensitivity and wide linear dynamic range. In addition to the TaqMan assay, the SYBR® Green PCR assay is another commonly used real-time PCR technique which is employed by half of all real-time PCR users. SYBR Green PCR is widely used because of the ease in designing the assays and its relatively low setup and running costs [19,20]. One drawback of SYBR Green assays, however, is that the dye is non-specific and can generate false positive signals if non-specific products or primer-dimmers are present in the assay [21]. Those problems can be addressed by carefully designing the primers and validating the PCR products with dissociation curve analysis immediately after PCR.

So the Real-time PCR Array System is the ideal tool for analyzing the expression of a focused panel of genes. The flexibility, simplicity, and convenience of standard SYBR Green PCR detection methodology make the PCR Array System accessible for routine use in any research laboratory [22]. The specificity of the system guarantees the amplification of only one gene-specific product in each reaction meaning that the expression level result confidently reflects only the gene of interest. The present study demonstrates SYBR Green Real-time PCR Arrays to be a quantitative platform with high inter run and inter-laboratory reproducibility. PCR Arrays produce gene profiling differences between the two RNA samples that are highly concordant with those generated by other quantitative gene expression analysis and microarray platforms. PCR Arrays deliver results comparable to those of high-density microarrays. Moreover, it yields results similar to those of TaqMan Gene Expression Assays, a widely accepted method for validating microarray results, and other more complicated and more expensive quantitative methods tested by the TaqMan assay [19].

In this study, we will analyze the dyes-regulation genes and pathways in pediatric AML with this powerful platform, Real-time PCR arrays.

## Results and discussion

### Design the Real-time PCR array

We designed and tested 88 real-time PCR primer pairs for a quantitative gene expression analysis of key genes involved in pediatric AML (Additional file 1). Briefly ,we assayed the expression of 11 genes of HOX family [HOXA1, HOXA3, HOXA4, HOXA5, HOXA7, HOXA9, HOXB6, HOXB7, HOXB9 and HOXB9], 15 apoptosis related genes [CASP1, CASP4, CASP8, BCL2, BIK, BIRC5, BAX, BCL2L1, S100A8, S100A9, ID1, ID2,ID3, ID4 and TNF], 7 chemokines [CCL5, CCR1, CCR2, CCR4, CCR5 and CXCR4 ], 13 tumor related genes [WT1, BRCA1 ,NF1, RB, APC, TP53BP1, PTEN, TP53 , CDKN1A, CDKN2B, CDKN1C, Jun and CCNB1] and 17 important genes in leukemia[ HMBS, CDH1, STAT4, TIMP1,CD44, CDC42, DDX1, DKK3, HMGB2, STMN1, STMN2, MEIS1, MEIS2, IGFBP3, IL6, LGALS4 and GSK3B]. Each gene was tested the expression analysis and melting curve analysis to make sure the primer is specific for the target gene. The average CV for the CT values generated from assays on the PCR Array is found to be 0.73% with replicate measurements for CT values below 30 within 0.20 cycle average standard deviation, demonstrating a good inter-run reproducibility.

### Expression profile analysis of pediatric AML and normal control samples

**Figure 1:**
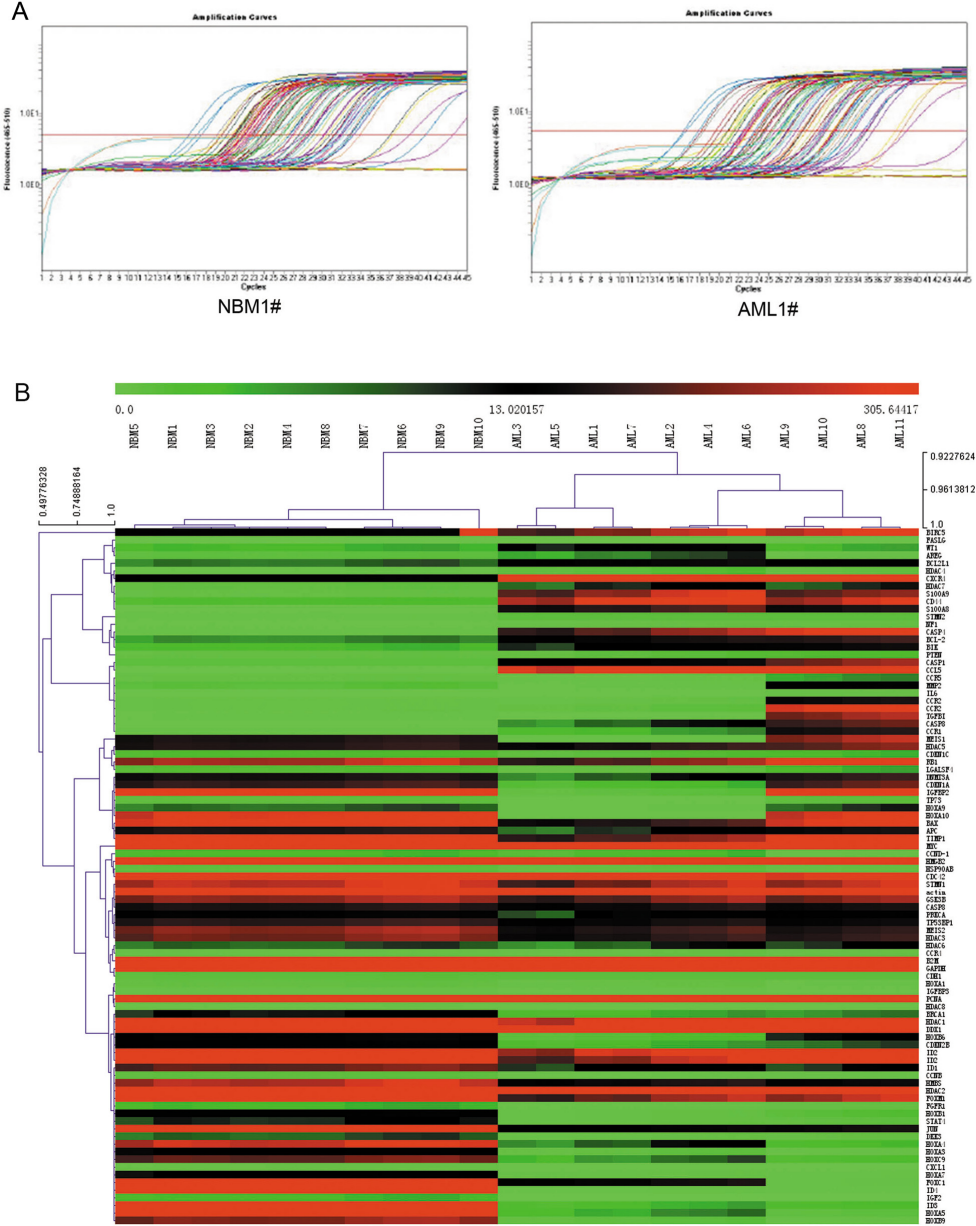
**Expression profile analysis of pediatric AML and normal control samples.** (**A**) Amplification data of Real-time PCR arrays in the NBM and pediatric AML. (**B**) Cluster analysis the data from Real-time PCR arrays.

**Figure 2:**
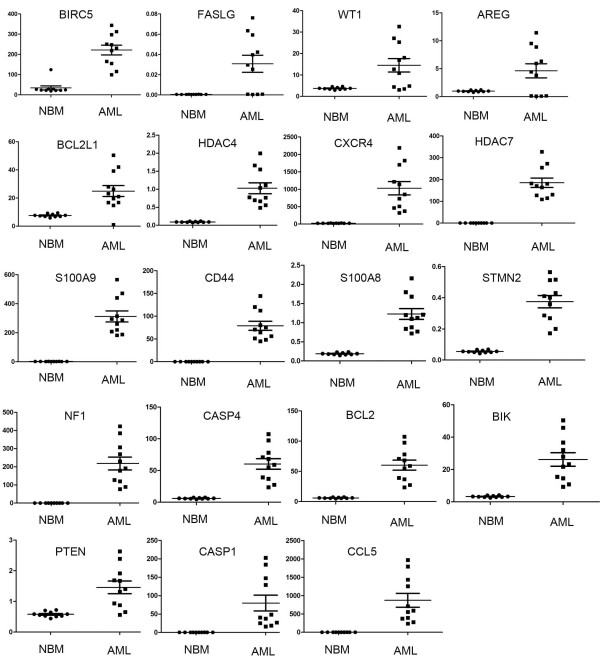
**Expression of up-regulated genes in pediatric AML.** The expression of the pediatric AML samples compared to the control samples was presented average ± SE. A *p* <0.05 was considered statistically significant.

**Figure 3:**
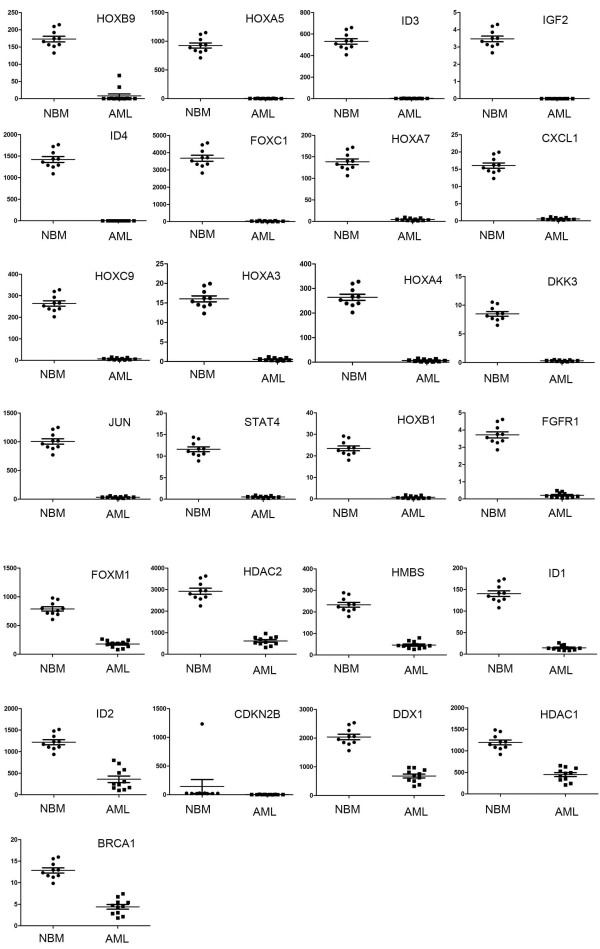
**Expression of down-regulated genes in pediatric AML.** The expression of the pediatric AML samples compared to the control samples was presented average ± SE. A *p* <0.05 was considered statistically significant.

We analyzed gene expression profile of pediatric AML and control samples with our Real-time PCR arrays (Figure 1A). The information of 10 normal control and 11 pediatric AML samples are listed in Table 1. After we get the original data, we analyzed the expression data with MEV (Multi Experiment View) cluster software. The gene expression profile of pediatric AML is significantly different from normal control, set of genes can be successfully clustered (Figure 1B). The results showed compared with normal control, there are 19 genes up-regulated and 25 genes down-regulated in pediatric AML (Table 2 and 3). The detailed expression of each up-regulated gene in pediatric AML was presented in Figure 2 and the expression of down-regulated genes was presented in Figure 3. Some of the dyes-regulated genes are consistent with other’s report, such as BIRC5, WT1, BCL2, S100A8 and CDKN2B. Oto et al. showed high expression of survivin in AML and survivn is a bad prognostic indicator in cases with acute leukemia especially in AML [23]. Barragan et al. showed that the Wilms' tumor (WT1) gene is over expressed in patients with most forms of acute leukemia. WT1 expression was significantly higher in AML patients than in normal controls (p =0.0001) [24]. Twenty-five patients with ALL and 65 patients with AML, both recently diagnosed, were included into a study. A high frequency of BCL2 mRNA over expression and a relatively low frequency of BAX mRNA over expression detected in both analyzed leukemia in this study, indicate that altered transcription of these genes may be involved in leukemogenesis [25]. Nicolas et al. used mass spectrometry (MS)-based proteomic approaches to characterize that S100A8 is up-regulated in leukemia cells and the expression of S100A8 in leukemic cells is a predictor of low survival [26]. CDKN2B appears to be frequently deleted and methylated in AML [27-30].

**Table 1:** Pediatric acute myeloid leukemia patients’ clinical feature

	**Age**	**Sex**	**Hb**	**WBC**	**RBC**	**PLt**	**Diagnosis**	**Chromosome**	**Fusion gene**
1	7	M	80	455. 8	2. 88	15	AML- M1	46,XY	HOX11
2	4	F	83	136. 1	3. 09	11	AML- M1	46,XY	ns
3	5	F	73	38. 83	2. 25	14	AML- M5b	46,XY,t ( 1, 11) ( q21; q23)	ns
4	9	M	45	2. 47	1. 49	10	AML- M3	46, XY,t ( 11; 21) , t ( 15; 17) [ 12] / 46, XY, [ 3]	PML/ RARA
5	0.9	F	76	56. 95	3. 52	155	AML- M4b	ns	MLL/ AF19
6	3	M	82	19. 58	2. 68	11	AML- M7	46,XY,t ( 2; 16)(q37; q12) , t ( 12, 20) ( p13, q11)	ns
7	0.9	F	73	323. 54	2. 74	33	AML- M4b	ns	ns
8	4	M	80	7. 97	2. 3	91	AML- M5	ns	AML/ ETO
9	11	M	80	35. 3	2. 45	75	AML- M3a	46, XY	PML/ RAR1
10	6	M	68	39. 4	2. 39	18	AML- M3	46,XY	PML/ RARA
11	7	F	54	4. 56	1. 75	17	AML- M3	46,XX,t ( 15; 17) ( q22; q21) [ 3] / 46,XX[ 12]	PML/ RARA

*Hb*: Hemoglobin *WBC*: White blood cells *RBC*: Red blood cells *PLt*: Platelet.

**Table 2:** Genes up regulated in the pediatric acute myeloid leukemia compared with normal control

	**Gene**	**Description**	**NBM**	**AML**	**Fold change**	**P**
1	BI RC5	Baculoviral IAP repeat containing 5,survivin	34. 0496	221. 4575	6. 504	<0. 0001
2	FASLG	Fas ligand (TNF superfamily, member 6)	0. 0005	0. 0308	57. 068	<0. 0001
3	WT1	Wilms tumor 1	3. 7431	14. 5000	3. 874	<0. 0001
4	AREG	Amphiregulin	0. 9960	4. 6307	4. 649	<0. 0001
5	BCL2L1	BCL2-like 1	7. 6435	27. 1093	3. 547	0. 0002
6	HDAC4	Histone deacetylase 4	0. 0924	1. 0273	11. 117	0. 0002
7	CXCR4	Chemokine (C-X-C motif) receptor 4	24. 1547	1029. 2052	42. 609	0. 0004
8	HDAC7	Histone deacetylase 7	0. 7191	11. 6169	16. 155	0. 0006
9	S100A9	S100 calcium binding protein A9	0. 4583	185. 0090	403. 722	0. 0007
10	CD44	CD44 molecule (Indian blood group)	2. 1648	312. 4866	144. 349	0. 0007
11	S100A8	S100 calcium binding protein A8	0. 0027	79. 0206	29736. 015	0. 0007
12	STMN2	Stathmin-like 2	0. 1887	1. 2260	6. 497	0. 0007
13	NF1	Neurofibromin 1	0. 0553	0. 3749	6. 775	0. 0009
14	CASP4	Caspase 4	0. 1491	218. 1356	1463. 137	0. 0009
15	BCL- 2	B-cell CLL/lymphoma 2	6. 1230	60. 3025	9. 849	0. 0009
16	BI K	BCL2-interacting killer (apoptosis-inducing)	3. 3040	26. 2063	7. 932	0. 001
17	PTEN	Phosphatase and tensin homolog	0. 5800	1. 4595	2. 516	0. 001
18	CASP1	Caspase 1	0. 3028	79. 9140	263. 884	0. 004
19	CCL 5	Chemokine (C-C motif) ligand 5	0. 0288	872. 0606	30237. 515	0. 004

*NBM*: Mean value of the gene expression in NBM group *AML*: Mean value of the gene expression in pediatric AML group.

**Table 3:** Genes down regulated in the pediatric AML compared with normal control

	**Gene**	**Description**	**NBM**	**AML**	**Fold Change**	**P**
1	HOXB9	Homeobox B9	172. 9526	0. 6802	0. 004	<0. 0001
2	HOXA5	Homeobox A5	925. 5920	3. 4967	0. 004	<0. 0001
3	I D3	I nhi bi t or of DNA bi ndi ng 3	531. 6130	3. 0212	0. 006	<0. 0001
4	I GF2	Insulin-like growth factor 2	3. 4683	0. 0056	0. 002	<0. 0001
5	I D4	I nhi bi t or of DNA bi ndi ng 4	1422. 5193	0. 1906	0. 000	<0. 0001
6	FOXC1	Forkhead box C1	3676. 7938	26. 5854	0. 007	<0. 0001
7	HOXA7	Homeobox A7	15. 3933	0. 2396	0. 016	<0. 0001
8	CXCL1	Chemokine (C-X-C motif) ligand 1	0. 1130	0. 0022	0. 020	<0. 0001
9	HOXC9	Homeobox C9	138. 5471	4. 6762	0. 034	<0. 0001
10	HOXA3	Homeobox A3	16. 0470	0. 5843	0. 036	<0. 0001
11	HOXA4	Homeobox A4	263. 9704	7. 3403	0. 028	<0. 0001
12	DKK3	Dickkopf 3 homolog	8. 4810	0. 3344	0. 039	<0. 0001
13	JUN	Jun pr ot o- oncogene	1005. 8731	35. 1804	0. 035	<0. 0001
14	STAT4	Signal transducer and activator of transcription 4	11. 5854	0. 5332	0. 046	<0. 0001
15	HOXB1	Homeobox B1	23. 4942	0. 8037	0. 034	<0. 0001
16	FGFR1	Fibroblast growth factor receptor 1	3. 7172	0. 2184	0. 059	<0. 0001
17	FOXM1	For khead box M1	789. 1920	177. 9791	0. 226	0. 0008
18	HDAC2	Histone deacetylase 2	2925. 0236	618. 9588	0. 212	0. 0008
19	HMBS	Hydr oxymet hyl bi l ane synt hase	233. 0075	46. 3417	0. 199	0. 0008
20	I D1	I nhi bi t or of DNA bi ndi ng 1	140. 4811	14. 8120	0. 105	0. 0008
21	I D2	I nhi bi t or of DNA bi ndi ng 2	1221. 3259	360. 7765	0. 295	0. 001
22	CDKN2B	Cyclin-dependent kinase inhibitor 2B	23. 9879	5. 1419	0. 214	0. 001
23	DDX1	DEAD ( Asp- Gl u- Al a- Asp) box hel i case 1	2039. 8291	681. 2089	0. 334	0. 001
24	HDAC1	Histone deacetylase 1	1196. 1914	449. 9266	0. 376	0. 002
25	BRCA1	Br east cancer 1, ear l y onset	12. 8548	4. 4127	0. 343	0. 004

*NBM*: Mean value of the gene expression in NBM group *AML*: Mean value of the gene expression in pediatric AML group.

This work also indicates some genes dyes-regulated in pediatric AML for the first time. FASLG, the protein encoded by this gene is the ligand for FAS. Interaction of FAS with this ligand is critical in triggering apoptosis of some types of cells such as lymphocytes. The Fas/FasL system as an important pathway inducing cell apoptosis participates in occurrence and development of leukemia. Leukemia cells generally are not sensitive or are resistant to Fas/FasL-mediated apoptosis, while it is one of important reasons resulting in immunoescape and unsensitivity of leukemia cells to chemotherapy. In recent years studies related to mechanisms of leukemia cell resistance to Fas/FasL-mediated apoptosis such as Fas and FasL mutation and expression abnormality, Fas signaling transduction pathway abnormality, and regulatory affect of apoptotic regulatory genes on Fas/FasL system, as well as strategies replying to antiapoptosis of leukemia cells including NF-kappa B, XIAP, membrane receptor CD28 and matrix metalloproteinase 7 obtained some progresses [31]. HDACs, this work showed HDAC4 and HDAC7 up-regulated, HDAC1 and HDAC2 down-regulated in pediatric AML. Recruitment of HDAC4 is necessary for PLZF-mediated repression in both normal and leukaemic cells [32]. Ectopic expression of PML recruits HDAC7 to PML NBs and leads to activation of MEF2 reporter activity [33]. HDACs 1 is critical in enhancing cytarabine-induced apoptosis in pediatric AML, at least partly mediated by Bim [34]. Evaluated the mRNA gene expression profile of 12 HDAC genes by quantitative real-time polymerase chain reaction in 94 consecutive childhood acute lymphoblastic leukaemia (ALL) samples and its association with clinical/biological features and survival. ALL samples showed higher expression levels of HDAC2, HDAC3, HDAC8, HDAC6 and HDAC7 when compared to normal bone marrow samples. HDAC1 and HDAC4 showed high expression in T-ALL and HDAC5 was highly expressed in B-lineage ALL [35]. And these results may indicate a different expression profile of histone deacetylases (HDACs) between pediatric ALL and AML. Histones play a critical role in transcriptional regulation, cell cycle progression, and developmental events. HDACs is common feature in several human malignancies and may represent an interesting target for cancer treatment, including hematological malignancies. This work also found 7 HOX genes down-regulated in pediatric AML. HOX gene transcription during definitive hematopoiesis is tightly regulated, but in a temporal manner [36]. In AML, increased expression of HoxB3, B4, A7–11 is found in the most primitive progenitors with expression of A7–11 aberrantly sustained in differentiating progenitors [37,38]. This study indicate an novel profile of HOX genes down-regulated in pediatric AML and these observations suggest that analyzing the expression profile of HOX genes would provide useful insights into pediatric myeloid leukemogenesis. Expression of HOX B6 and HOX B9 in NB4 and HL-60cells increase at a mid stage of myeloid differentiation by ATRA induction and then decrease during a late stage [39]. The phenotypic survey of Hoxa5 mutant mice has unveiled the crucial role of this gene in regulating morphogenesis and specifying regional identity along the embryo. A majority of Hoxa5 mutant pups die at birth from defective respiratory tract. Surviving mutants present deficient alveolar septation revealing the importance of Hoxa5 during formation and maturation of the lung. The implication of Hoxa5 in tumorigenesis has also been documented, the loss of Hoxa5 function limits leukaemia associated with specific chromosomal translocations. Thus, inappropriate Hoxa5 gene expression may disrupt normal growth and differentiation programs causing neoplasia [40]. Hypermethylation of HOXA5 is a good prognostic factor of AML patients. The patients of the AML group who had high methylation percentage had a good prognosis with a 3-yr overall survival. Cox proportional hazards regression showed that the methylation percentages of HOXA5 were independently associated with the 3-year overall survival of AML patients [36]. HOXA4 gene expression is a predictor for outcome in normal karyotypic AML patients. 77% AML patients with HOXA4 hypermethylated and the low HOXA4 expression is a favourable predictor for outcome in AML patients [41].

### Ingenuity pathway analysis the dyes-regulated genes in pediatric AML

**Figure 4:**
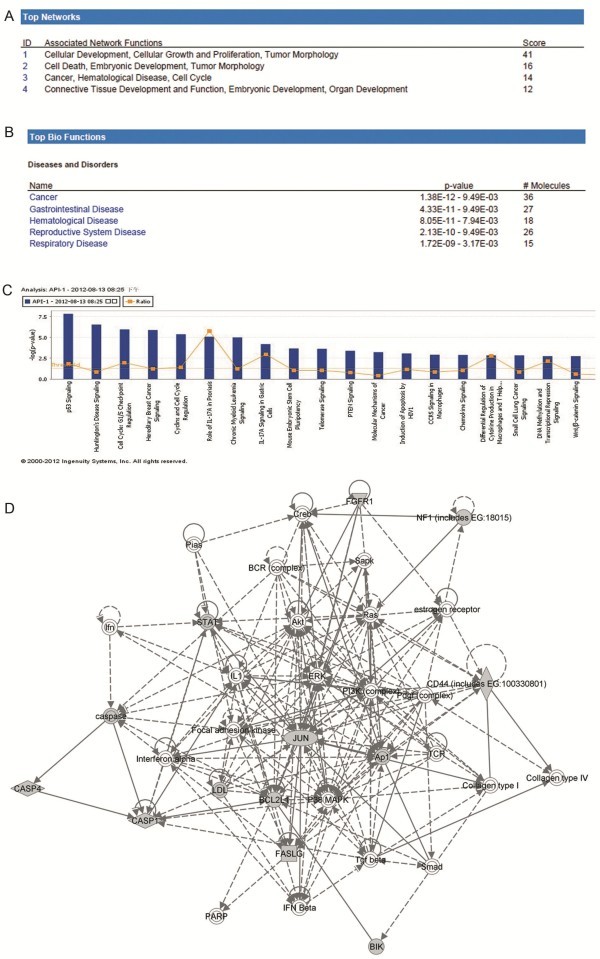
**Ingenuity Pathways Analysis (IPA) summary.** (**A**) The list of top four networks with their respective scores obtained from IPA. (**B**) The list of top five bio functions with their respective scores obtained from IPA. (**C**) Toxicology pathway list in IPA analysis. The x-axis represents the top toxicology functions as calculated by IPA based on differentially expressed genes are highlighted and the y-axis represents the ratio of number of genes from the dataset that map to the pathway and the number of all known genes ascribed to the pathway. The yellow line represents the threshold of p value, 0.05 as calculated by Fischer’s test. (**D**) Most highly rated network in IPA analysis. The network representation of the most highly rated network. The genes that are shaded were determined to be significant from the statistical analysis. A solid line represents a direct interaction between the two gene products and a dotted line means there is an indirect interaction.

To investigate possible biological interactions of differently regulated genes, datasets representing genes with altered expression profile derived from real-time PCR array analyses were imported into the Ingenuity Pathway Analysis Tool. The list of differentially expressed genes analyzed by IPA revealed 12 significant networks. Figure 4A represents the list of top 4 networks identified by IPA. Of these networks, Cellular Development, Cellular Growth and Proliferation, Tumor Morphology was the highest rated network with 36 focus molecules and the significance score of 41 (Figure 4D). The score is the probability that a collection of genes equal to or greater than the number in a network could be achieved by chance alone. A score of 3 indicates a 1/1000 chance that the focus genes are in a network not due to random chance.

The IPA analysis also groups the differentially expressed genes into biological mechanisms that are related to cancer groups, hematological disease, cell death, cell growth and proliferation, cardiovascular system development and function, tumor morphology and hematological system development and function (Figure 4B). In the toxicology list, p53 and Huntington’s disease signaling came out to be the top two most significant pathways with a p value of 1.5E-8 and2.95E-7, respectively (Figure 4C). The genes associated with the top toxicology list are also given in the Additional file 2.

This IPA analysis showed in pediatric AML the top important pathways are p53 and Huntington’s disease signaling. P53 protein expression has been widely investigated in leukemia and there are hundreds of papers about the important roles of p53 in the pediatric leukemia [42-50]. But there is still no report about the relationship between Huntington’s disease signaling and AML. This work may provide new clues of molecular mechanism in pediatric AML.

## Conclusions

The present study demonstrates the gene expression profile of pediatric AML is significantly different from normal control; there are 19 genes up-regulated and 25 genes down-regulated in pediatric AML. We found some genes dyes-regulated in pediatric AML for the first time as FASLG, HDAC4, HDAC7 and some HOX family gene. IPA analysis showed the top important pathways for pediatric AML are p53 and Huntington’s disease signaling. This work may provide new clues of molecular mechanism in pediatric AML.

## Methods

### Patients and samples

Bone marrow specimens were obtained at the time of diagnosis during routine clinical assessment of 11 patients with AML, who presented at the Department of Hematology and Oncology, Children's Hospital of Soochow University between 2011 and 2012. Ethical approval was provided by the Children's Hospital of Soochow University Ethics Committee (No. SUEC2010-011), and informed consent was obtained from the parents or guardians. AML diagnosis was made in accordance with the revised French–American–British (FAB) classification. The main clinical and laboratory features of the patients cohort are summarized in Table 1. Additionally, bone marrow samples from 10 healthy donors were analyzed as controls. Bone marrow mononuclear cells (BMNCs) were isolated using Ficoll solution within 2 h after bone marrow samples harvested and immediately subjected for the extraction of total RNA.

### RNA extraction

For RNA extraction, bone marrow samples were immediately submerged in 2 ml Trizol (Invitrogen), stored at −80°C until further processed. A volume of 1 ml of each sample was spun at 4°C for 15 min at 12,000 g to remove debris and DNA, 1 ml of supernatant was mixed with 200 ul chloroform, shaken for 15 seconds, incubated at RT for 2–3 minutes and spun for 10 min at 12,000 g at 4°C. RNA was precipitated by adding 500 ul of the aqueous phase to an equal volume of isopropanol and spun at 14,000 g at 4°C for 10 min. RNA was washed with 75% ethanol, spun at 14,000 g at 4°C for 10 min, dried and resuspended in 40 ul DEPC-treated H2_O_. The final RNA concentration was determined using a spectrophotometer (Nanodrop 2000) and the purity was assessed by agarose gel electrophoresis.

### CDNA synthesis

CDNA synthesis was performed on 4 ug of RNA in a 10 ul sample volume using SuperScript II reverse transcriptase (Invitrogen) as recommended by the manufacturer. The RNA was incubated with 0.5 ug of oligo (dT) 12–18mers primers (Invitrogen) for 7 min at 70°C and then transferred onto ice. Then, 9 ul of a master mix containing 4 ul of SuperScript II buffer, 2 ul of 0.1 M DTT (Invitrogen), and 1 ul each of dNTPs stock (10 mM) (Invitrogen), Rnasin (40 UI) (Promega) and SuperScript II (Invitrogen) were added to the RNA sample, spun and incubated at 42°C for 60 min followed by 5 min at 70°C to inactivate the enzyme. CDNA was stored at −20°C.

### Real-time PCR array design and test

Most of the primers were from a database of Real-time primers, Center for Medical Genetics (http://medgen.ugent.be/CMGG/). The rest of primers were designed using the online program Primer 3 (http://www.fokker.wi.mit.edu/primer3/input.htm). Primer selection parameters were set to primer size: 20–26 nts; primer melting temperature: 60 to 64°C; GC clamp: 1; and product size range: generally 120–240 bp but down to 100 bp if no appropriate primers could be identified. Primers were ordered from Invitrogen. (Genes and sequence of the primers was presented in Additional file 1).

### Real-time PCR array analysis

Real-time PCR array analysis was performed in a total volume of 20 ul including 2ul of cDNA, primers (0.2 mM each) and 10 ul of SYBR Green mix (Roche). Reactions were run on an Light cycler 480 (Roche) using the universal thermal cycling parameters (95°C 5 min, 45 cycles of 10 sec at 95°C, 20 sec at 60°C and 15 sec at 72°C; melting curve: 10 sec at 95°C, 60 sec at 60°C and continues melting). Results were obtained using the sequence detection software Light cycler 480 and analyzed using Microsoft Excel. For all samples melting curves were acquired for quality control purposes. For gene expression quantification, we used the comparative Ct method. First, gene expression levels for each sample were normalized to the expression level of the housekeeping gene encoding Glyceraldehydes 3-phosphate dehydrogenase (GAPDH) within a given sample (−⊿Ct); the relative expression of each gene was calculated with 10^6^ *Log_2_(−⊿Ct ).The difference between the pediatric AML samples compared to the control samples was used to determine the10^6^ *Log_2_(−⊿Ct ). Statistical significance of the gene expression difference between the AML and the control samples was calculated with the T-test using SPSS 11.5 software.

### Ingenuity pathway analysis (IPA)

Datasets representing genes with altered expression profile derived from Real-time PCR array analyses were imported into the Ingenuity Pathway Analysis Tool (IPA Tool; Ingenuity H Systems, Redwood City, CA, USA; http://www.ingenuity.com). In IPA, differentially expressed genes are mapped to genetic networks available in the Ingenuity database and then ranked by score. The basis of the IPA program consists of the Ingenuity Pathway Knowledge Base (IPKB) which is derived from known functions and interactions of genes published in the literature. Thus, the IPA Tool allows the identification of biological networks, global functions and functional pathways of a particular dataset. The program also gives the significance value of the genes, the other genes with which it interacts, and how the products of the genes directly or indirectly act on each other, including those not involved in the microarray analysis. The networks created are ranked depending on the number of significantly expressed genes they contain and also list diseases that were most significant. A network is a graphical representation of the molecular relationships between molecules. Molecules are represented as nodes, and the biological relationship between two nodes is represented as an edge (line). All edges are supported by at least 1 reference from the literature, from a textbook, or from canonical information stored in the Ingenuity Pathways Knowledge Base.

### Statistical analysis

SPSS v11.5 (SPSS Inc., Chicago, IL) was used for statistical analysis. For gene expression quantification, we used the comparative Ct method. First, gene expression levels for each sample were normalized to the expression level of the housekeeping gene encoding Glyceraldehydes 3-phosphate dehydrogenase (GAPDH) within a given sample (−⊿Ct); the relative expression of each gene was calculated with 10^6^ *Log_2_(−⊿Ct ).The expression of the pediatric AML samples compared to the control samples was presented average ± SE. A *p*<0.05 was considered statistically significant.

## Competing interests

The authors have no conflicts of interest to disclose.

## Authors’ contributions

PJ designed and directed the study. TYF finished the most of the experiments. WJ, FX and LYH, coordinated data collection and quality control, and assisted in the interpretation of results. WD, PL, ZWL, LJ and WN participated in acquiring laboratory data analysis. NJ participated in study design and coordination, data analysis and interpretation and drafted the manuscript. All authors read and approved the final manuscript.

## Supplementary Material

Additional file 1 Genes and PCR primers of Real-time PCR array. Click here for file

Additional file 2 Summary of IPA analysis. Click here for file
