# Supplementary material for: Analyzing the gene expression profile of pediatric acute myeloid leukemia with real-time PCR arrays
Source: Cancer Cell Int. 2012 Sep 8;12:40. doi: 10.1186/1475-2867-12-40 (PMC3495223; doi:10.1186/1475-2867-12-40)
Supplement: Additional file 2 — Summary of IPA analysis. [file 1475-2867-12-40-S2.pdf]

IPA® Summary of Analysis

Analysis Name: API-1 - 2012-08-13 08:25
Analysis Creation Date: 2012-08-13
Build version: 162830
Content version: 12710793 (Release Date: 2012-05-07)

Analysis settings

View

Reference set: Ingenuity Knowledge Base (Genes Only)

Relationship to include: Direct and Indirect

Includes Endogenous Chemicals

Optional Analyses: My Pathways My List

Filter Summary:

Consider only molecules and/or relationships where

(species = Human OR Uncategorized (e.g. chemicals)) AND

(confidence = Experimentally Observed) AND

(tissues/cell lines = Retina OR THP-1 OR Skeletal Muscle OR Immature monocyte-derived dendritic cells OR Large Intestine OR Vd2 Gamma-delta T cells OR Jurkat OR MOLT-4 OR Monocyte-derived macrophage OR Naive B cells OR Pancreas OR Murine NKT cells OR Naive helper T cells OR Vd1 Gamma-delta T cells OR Central memory helper T cells OR CD56bright NK cells OR Activated CD56bright NK cells OR Prostate Gland OR Heart OR SR OR Activated helper T cells OR RAW 264.7 OR Placenta OR Ovary OR Kidney OR Spleen OR HL-60 OR Activated Vd1 Gamma-delta T cells OR Adipose OR Natural T-regulatory cells OR Effector memory cytotoxic T cells OR Thymus OR Activated CD56dim NK cells OR Lung OR Liver OR Small Intestine OR Uterus OR Th2 cells OR Effector memory helper T cells OR Other Tissues and Primary Cells OR Activated Vd2 Gamma-delta T cells OR CD56dim NK cells OR Macrophages OR Monocytes OR Central memory cytotoxic T cells OR Mature monocyte-derived dendritic cells OR Memory B cells OR Bladder OR Stomach OR CCRF-CEM OR K-562 OR BDCA-1+ dendritic cells OR

(c) 2000-2012 Ingenuity Systems, Inc. All rights reserved.

Cytotoxic T cells OR Effector memory RA+ cytotoxic T cells OR Neutrophils OR Effector T cells OR Salivary Gland OR Mammary Gland OR BDCA-3+ dendritic cells OR Th1 cells OR Epidermis OR Testis OR Plasmacytoid dendritic cells) AND
(data sources = BIND OR BIOGRID OR Cognia OR DIP OR Ingenuity Expert Findings OR Ingenuity ExpertAssist Findings OR INTACT OR Interactome studies OR MINT OR MIPS OR TarBase)

Top Networks

ID	Associated Network Functions	Score
1	Cellular Development, Cellular Growth and Proliferation, Tumor Morphology	41
2	Cell Death, Embryonic Development, Tumor Morphology	16
3	Cancer, Hematological Disease, Cell Cycle	14
4	Connective Tissue Development and Function, Embryonic Development, Organ Development	12

(c) 2000-2012 Ingenuity Systems, Inc. All rights reserved.

INGENUITY® SYSTEMS

Top Bio Functions

Diseases and Disorders

Name	p-value	# Molecules
Cancer	1.38E-12 - 9.49E-03	36
Gastrointestinal Disease	4.33E-11 - 9.49E-03	27
Hematological Disease	8.05E-11 - 7.94E-03	18
Reproductive System Disease	2.13E-10 - 9.49E-03	26
Respiratory Disease	1.72E-09 - 3.17E-03	15

Molecular and Cellular Functions

Name	p-value	# Molecules
Cell Death	5.92E-14 - 9.49E-03	25
Gene Expression	1.63E-13 - 9.49E-03	27
Cellular Development	3.29E-12 - 9.49E-03	27
Cellular Growth and Proliferation	1.06E-11 - 9.49E-03	24
Cellular Movement	9.58E-11 - 9.49E-03	19

Physiological System Development and Function

Name	p-value	# Molecules
Cardiovascular System Development and Function	5.68E-08 - 8.93E-03	11
Organismal Development	5.68E-08 - 9.49E-03	11
Tumor Morphology	8.86E-08 - 9.49E-03	15
Embryonic Development	4.79E-06 - 9.49E-03	13
Hematological System Development and Function	6.96E-06 - 6.76E-03	16

(c) 2000-2012 Ingenuity Systems, Inc. All rights reserved.

Top Canonical Pathways

Name	p-value	Ratio
p53 Signaling	1.5E-08	7/96 (0.073)
Huntington's Disease Signaling	2.95E-07	8/233 (0.034)
Cell Cycle: G1/S Checkpoint Regulation	1.12E-06	5/64 (0.078)
Hereditary Breast Cancer Signaling	1.32E-06	6/123 (0.049)
Cyclins and Cell Cycle Regulation	4.22E-06	5/87 (0.057)

Top Molecules

This analysis has no expression values.

Top Upstream Regulators

Upstream Regulator	p-value of overlap	Predicted Activation State
phorbol myristate acetate	3.14E-14	
EGF (includes EG:13645)	1.83E-13	
dexamethasone	1.83E-13	
TP53 (includes EG:22059)	8.04E-12	
Akt	3.14E-11	

(c) 2000-2012 Ingenuity Systems, Inc. All rights reserved.

Top My Lists

Name	p-value	Ratio
Direct players of IL24	1.8E-03	2/21 (0.095)
Cell Differentiation	1.47E-01	1/50 (0.02)
BIOMARKERS HNC and mouse HPV16	2.71E-01	1/100 (0.01)

Top My Pathways

Name	p-value	Ratio
FASN malignancy pathway	1.76E-04	4/143 (0.028)
IL24 pic	4.66E-04	3/54 (0.056)
Networks 13,3 Merged 2	2.82E-03	2/50 (0.04)
Merged networks	2.42E-02	2/268 (0.007)

Top Tox Lists

Name	p-value	Ratio
p53 Signaling	1.74E-08	7/95 (0.074)
Cell Cycle: G1/S Checkpoint Regulation	1.21E-06	5/60 (0.083)
Renal Necrosis/Cell Death	1.81E-06	9/368 (0.024)
Liver Proliferation	2.32E-05	6/184 (0.033)
Cardiac Necrosis/Cell Death	2.62E-05	6/188 (0.032)

(c) 2000-2012 Ingenuity Systems, Inc. All rights reserved.

INGENUITY® SYSTEMS

Top Tox Functions

Cardiotoxicity

Name	p-value	# Molecules
Cardiac Infarction	6.34E-03 - 6.34E-03	1
Cardiac Necrosis/Cell Death	1.75E-02 - 1.75E-02	2
Pulmonary Hypertension	1.03E-01 - 1.03E-01	1
Cardiac Hypertrophy	1.55E-01 - 1.55E-01	1

Hepatotoxicity

Name	p-value	# Molecules
Hepatocellular Carcinoma	1.29E-04 - 4.66E-02	8
Liver Hyperplasia/Hyperproliferation	1.29E-04 - 4.66E-02	8
Liver Necrosis/Cell Death	1.38E-04 - 3.44E-02	3
Liver Cirrhosis	1.18E-03 - 2.60E-02	4
Liver Degeneration	3.17E-03 - 3.17E-03	1

Nephrotoxicity

Name	p-value	# Molecules
Renal Necrosis/Cell Death	1.62E-05 - 1.89E-02	9
Glomerular Injury	3.17E-03 - 3.17E-03	1
Kidney Failure	3.17E-03 - 3.17E-03	1
Renal Proliferation	4.94E-03 - 7.05E-02	4
Renal Inflammation	4.66E-02 - 4.66E-02	1

(c) 2000-2012 Ingenuity Systems, Inc. All rights reserved.
